# Prevalence of Bovine Viral Diarrhoea Virus (BVDV), Bovine Herpes Virus 1 (BHV 1), Leptospirosis and Neosporosis, and associated risk factors in 161 Irish beef herds

**DOI:** 10.1186/s12917-017-1324-9

**Published:** 2018-01-06

**Authors:** Damien Barrett, Mervyn Parr, John Fagan, Alan Johnson, Jamie Tratalos, Francis Lively, Michael Diskin, David Kenny

**Affiliations:** 1 Department of Agriculture, Food and the Marine, SAT Division, Admin Building, Backweston, Celbridge, Co. Kildare Ireland; 2 Department of Agriculture, Food and the Marine, Athlone RVL, Co. Westmeath, Athlone, Ireland; 3 Department of Agriculture, Food and the Marine, Limerick RVL, Limerick, Co. Limerick, Ireland; 4 Teagasc, Grange, Dunsany, Trim, Co. Meath, Ireland; 5 Teagasc, Mellows College, Athenry, Co. Galway Ireland; 6 Centre for Veterinary Epidemiology and Risk Analysis, University College Dublin, Belfield, Dublin 4, Ireland; 7 Agri-Food and Biosciences Institute, 18a Newforge Lane, Belfast, Co Antrim BT9 5PX Northern Ireland

**Keywords:** Beef cow herds, BVDV, BHV-1, Neosporosis, Leptospirosis, Herd size, Pathogen associations

## Abstract

**Background:**

There are limited data available, in Ireland or elsewhere, to determine the extent of exposure to various endemic diseases among beef cows and factors associated with exposure to causative pathogens. The objectives of this study were to determine the herd and within herd prevalence of Bovine Viral Diarrhoea Virus **(**BVDV), Bovine Herpes Virus 1 **(**BHV-1), Leptospirosis and Neosporosis in a large scale study of commercial beef herds on the island of Ireland, and to examine herd level factors associated with exposure to these pathogens in these herds.

**Results:**

The average number of cows tested per herd was 35.5 (median 30). Herd level seroprevalence to Bovine Herpesvirus-1(BHV-1), Bovine Viral-Diarrhoea Virus (BVDV), Leptospirosis and Neosporosis was 90%, 100%, 91% and 67%, respectively, while the mean within herd prevalence for the these pathogens was 40%, 77.7%, 65.7% and 5.7%, respectively. The study confirms that the level of seroconversion for the four pathogens of interest increases with herd size. There was also evidence that exposure to one pathogen may increase the risk of exposure to another pathogen.

**Conclusions:**

Herd level seroprevalences were in excess of 90% for BVDV, BHV-1 and Leptosporosis. Larger herds were subject to increased exposure to disease pathogens. This study suggests that exposure to several pathogens may be associated with the further exposure to other pathogens.

## Background

In recent years, the issue of herd health and, in particular, infectious disease has received increasing prominence as a potential factor influencing reproductive efficiency in Ireland and elsewhere. However, despite its obvious critical importance, there are limited published data available, to quantify the influence of various endemic diseases on beef cow and herd fertility. Reproductive performance in beef cow herds is related to several management, physiological and disease factors [1, 2]. The current level of reproductive performance in Irish beef cow herds leave considerable room for improvement in achieving more efficient, sustainable and profitable beef cow beef production [3]. There is a need to establish the prevalences of the most significant infectious diseases associated with impaired reproductive performance.

Numerous bacterial, viral and protozoan pathogens have been associated with infertility and abortion in cattle. BVDV, BHV-1, *Leptospira interrogans* hardjo-bovis and *Neospora caninum* are four of the most significant pathogens associated with infertility and abortion in Irish cattle. While non-infectious agents probably contribute more to overall reproductive wastage in cattle, there is limited information on the extent and importance of infectious disease on reproductive performance in Irish beef cow herds.

BVDV is a pestivirus which is endemic in the cattle population of several countries and is responsible for a wide range of clinical syndromes, amongst which are immunosuppression and early embryonic death [4]. In recent years, several countries including the Republic of Ireland and Northern Ireland have initiated national BVD eradication schemes. Data from the national BVD eradication scheme in the Republic of Ireland (initiated in 2012) reveal the presence of one or more BVD virus positive animals is 8.74%, 5.96% and 3.311%, 2.38% of beef herds for the years 2013, 2014, 2015 and 2016, respectively (Graham, personal communication). A recent study showed BVDV seroprevalence of 98% in Irish beef cow beef herds [5]. Furthermore, in a study commissioned by Animal Health Ireland prior to the launch of a national BVD eradication scheme, BVDV was estimated to cost the Irish beef cow industry between €32 and €41 per cow per year [6].

Bovine herpes virus 1 (BHV-1) is associated with clinical syndromes such as rhinotracheitis, pustular vulvovaginitis and balanoposthitis, abortion, infertility, conjunctivitis and encephalitis in cattle [7, 8]. It establishes latent lifelong infection and becomes reactivated during periods of immunosuppression, induced by stressful incidents, resulting in virus excretion when infection becomes re activated [9]. Two recent studies conducted on Irish beef [10, 11] and dairy herds [11] both reported herd level seroprevalence of BHV-1 to be approximately 75% and that this was consistent throughout the country.

Leptospirosis is a bacterial disease of cattle that is characterised by reproductive failure in the form of abortions, stillbirths and birth of weak calves. It is also a zoonotic disease. In cattle, the leptospires of most interest are interrogans hardjo-bovis and -prajitno genotypes (i.e. sub-serovars). A number of studies have reported reduced conception rates in seropositive dairy cows [12, 13].A previous study of Irish beef cow herds has shown that 82% of herds were seropositive for leptospirosis, with 40% of cows within those herds seropositive for leptospirosis [14].

Abortions due to *Neospora caninum* typically occur during the early second trimester, but may occur throughout gestation. A number of authors have suggested that cattle infected with the parasite are two to 12 times more likely to abort compared with uninfected cattle [15, 16]. These can be sporadic, endemic or epidemic-abortions [17]. In a large scale Canadian study, cows seropositive for Neosporosis were 1.43 times more likely to be culled for reproductive inefficiency than seronegative cows [18]. A recent Australian study of Neosporosis in beef cow herds reported that calving rates were 10.4% lower in herds that had one or more animals positive for Neosporosis [19] As far as the authors of this paper are aware, this is the first serological survey for Neosporosis in Irish beef cow herds.

The objectives of this study were to (i) determine the herd and within herd prevalence of BVDV, BHV-1, Leptospirosis and Neosporosis in a large cohort of commercial beef herds on the island of Ireland, and (ii) examine herd level factors associated with exposure to these pathogens in these herds.

## Methods

### Herd selection and sampling method

In all, 6049 cows in 161 beef cow herds from across the island of Ireland were sampled in this study. Of these, 156 were commercial beef cow herds, including 25 participating in national extension programmes. The remaining five herds were located on state owned research facilities. Blood sampling took place once for each herd over the summer months of either 2014 or 2015, and only spring calving cows within the herds were sampled. Data on herd vaccination and mineral supplementation policies were gathered at the time of sampling.

### Ethical statement on blood sampling

All experimental procedures involving animals were licensed by the Irish Health Products Regulatory Authority (HPRA). Protocols were conducted in accordance with the Cruelty to Animals Act (Ireland 1876, as amended by European Communities regulations 2002 and 2005) and the European Community Directive 86/609/EC and were sanctioned by the Teagasc Animal Ethics Committee. Blood samples were collected via the coccygeal vessel into 10 ml non additive containers (Becton Dickenson Vacutainer Systems, Plymouth, UK) for serological analysis.

### Laboratory processing

Sera were centrifuged and aliquots for each test were partitioned into serum vials and frozen at −20 °C pending analysis. Sera were screened using commercially available ELISA kits for BVDV, BHV-1, Leptospirosis and Neosporosis in Department of Agriculture, Food and the Marine Regional Veterinary Laboratory service (RVL). In each case the tests were carried out according to the manufacturer’s instructions.

For herds where IBR vaccination was reported, the kit used for BHV-1 was the IDEXX IBR gE kit (IDEXX, Maine, USA). The manufacturer’s report a sensitivity of 98.41% and a specificity of 99.76%. The cut off for a positive sample was an SN (sample to negative control ratio) value of <0.6.

In herds that did not declare an IBR vaccination policy the testing kit used for BHV-1 was the IDEXX IBR gB X3 (IDEXX, Maine, USA). The cut off for a positive sample for this kit was >55% blocking. The manufacturer’s report a sensitivity of 100% and a specificity of 99.8%.

The kit used for BVDV antibody was the Svanovir BVD – Ab Bovine Virus Diarrhoea Virus antibody Test (Boehringer Ingelheim Svanova, Uppsala, Sweden). The cut off for a positive result was a PP (percent positivity) value >35. The manufacturer’s report a sensitivity of 100% and a specificity of 98.2%.

The kit used for Leptospirosis antibody was the PrioCHECK *L.hardjo* antibody kit (Thermofischer, Massachusetts, USA). The manufacturer’s report a sensitivity of 99% and a specificity of 85%. The cut off for a positive sample was a PP (percent positivity) value of >45%.

The kit used for Neosporosis antibody was the IDEXX Neospora antibody kit (IDEXX, Maine, USA). The manufacturer’s report a sensitivity of 97.56% and a specificity of 98.53%. The cut off for a positive sample was a S/P (sample to positive ratio) value of >0.4.

### Herd level BVDV status

The herd level BVDV status for each of the Republic of Ireland herds (*n* = 139) was accessed from the Irish Cattle Breeding Federation (ICBF) database for the years 2013, 2014 and 2015. This information was not available for the herds located in Northern Ireland.

### Data management and analysis

Data were collated on Microsoft Excel spreadsheets (Microsoft Corporation, Redmond, WA, USA). Details of the herd demographics for Republic of Ireland herds are presented in Table 1. Only serological data and vaccination history were available for Northern Ireland herds. Each farmer was asked to complete a short questionnaire on vaccination and mineral supplementation practices in their herd. The vaccination status of the herd was based on the recall of the owner when completing the questionnaire at the time of sampling. We therefore cannot be certain that vaccinations were carried out in full compliance with the manufacturers’ instructions. However, where the farmer reported using a BVDV or Leptospirosis vaccine, these herds were removed from the analysis, in order to ensure, in as much as possible, that the analysis only related to naturally occurring infections. In the case of IBR / BHV-1, only marker vaccines have been available in the Republic of Ireland since 2005, and herds using IBR vaccination were tested with the marker vaccine test kit. Both marker and conventional vaccines are available for purchase in Northern Ireland and this may account for some of the seroconversion seen in the NI herds, as the tests we had available could not differentiate between seroconversion due to natural infection and seroconversion due to the use of a non-marker vaccine.

**Table 1:** Mean and median total herd size, number of cows, overall mortality rate, calf mortality rate up to 30 days, the number of calves per cow per year and purchase rate among Republic of Ireland herds in the study

Region	Herd Size		No cows		Mortality Rate		Calf Mortality rate up to 30 days		Calves per cow per year		Purchase rate	
	Mean	Median	Mean	Median	Mean	Median	Mean	Median	Mean	Median	Mean	Median
1	88	79	46.9	41.5	5.3	5.4	2.8	2	0.98	1	16.2	16.6
2	78	67	33.9	32.5	4.2	3.1	2.2	2	0.87	0.97	17.5	13.8
3	190	134	74.5	54	4.5	3.6	4.9	4.23	0.85	0.99	15.8	15.6
4	131	116	42.7	43	3.4	2.1	4.1	3.3	0.93	1	26.7	13.5
5	158	136	58.4	49	3.6	3.3	4.6	3.6	0.95	1	20.5	16.3
6	172	135	57.4	53.5	3	2.9	3.4	2.9	0.86	1	15.9	13.1
Overall	145	114	53.8	46	3.7	3.2	3.7	2.7	0.9	1	18.6	13.85

**Fig. 1:**
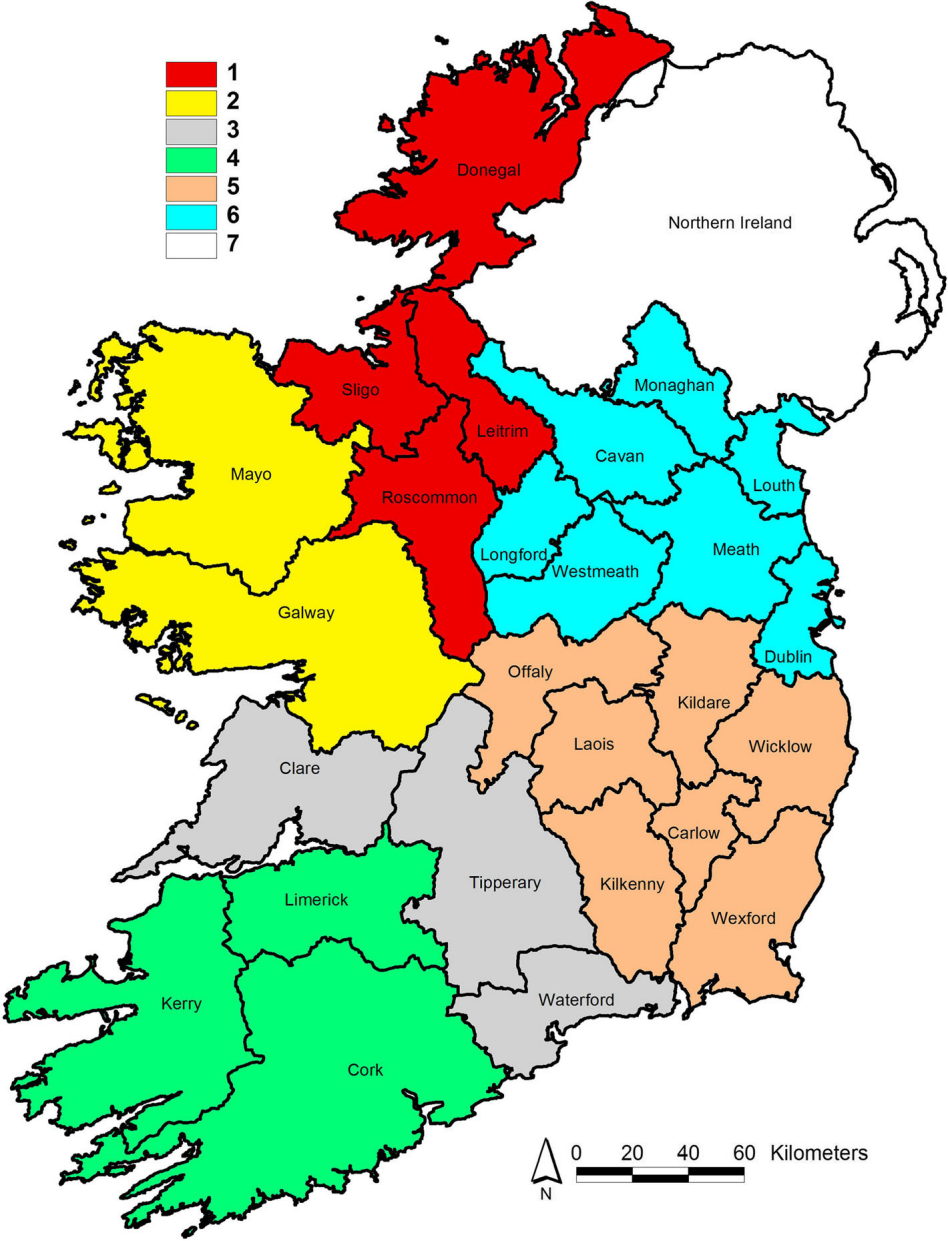
Geographical spread of the regions employed in the study

Herd level registration and movement data were obtained from the Irish Department of Agriculture, Food and the Marine’s Animal Identification and Movement (AIM) database and the following variables were calculated for each herd: herd size, number of cows, overall mortality rate, calf mortality up to 30 days of age, calves per cow per year and purchase rate. These variables and herd level prevalence of the various serological tests were calculated and entered into the spreadsheet, in addition to herd vaccination history. Statistical analysis was carried out using Stata 14 (StataCorp LP, Texas, USA). A descriptive statistical analysis of the data was initially conducted. In order to examine the potential for regional differences the herds were divided into seven geographical regions (Fig. 1) as described by Ryan et al. [14].

#### Relationship between pathogen and within herd seroprevalence

A univariate linear regression analysis was initially carried out using a range of herd level statistics downloaded from the AIM system and with the herd prevalence of each pathogen as the dependent variable of interest. Variables with a *P* value less than 0.2 were then considered for inclusion in a multivariate linear regression model for each pathogen (Tables 4, 6 and 9 and 11). Variables with a P value >0.05 were removed from the model in a stepwise fashion, until the variables remaining in the final model all reached a *P* value <0.05. For Leptospirosis and BVDV data from only non-vaccinating herds were included in the statistical model.

#### Logistic regression for BVD eradication scheme herd status

In order to identify independent variables for inclusion in a multivariate model for BVDV, univariate logistic regressions was calculated. The dependent variable was the disclosure of BVDV positive calves in the course of the national BVDV eradication scheme, from the inception of the scheme until 2015. Variables with a *P* value less than 0.2 were considered for inclusion in the multivariate model. Variables with a P value >0.05 were removed from the model in a stepwise fashion, until the variables remaining in the final model had a P value <0.05, as outlined for the linear regression analysis above.

## Results

There were 14, 18, 16, 17, 48, 26 and 22 herds in Regions 1 to 7 respectively. The mean total herd size was 145.0, which varied from 82.4 (median 70.0) in Region 1 to 172.0 (median 135.0) in Region 4 (Table 1). The mean number of calves born per herd (which was a proxy for the number of breeding cows in the herd) was 53.8 overall (median 46), and similar to the herd size varied from 33.9 (median 36.0) (Region 1) up to 58.4 (median 49.0) (Region 3). The average number of cows tested per herd was 35.5 (median 30). The mean level of overall herd mortality was 3.7% (median 3.2%), ranging from 4.7% (median 3.8%) % in Region 1 to 3.0% (median 2.9%) in region 4. Although the mean level of calf mortality up to 30 days of age was also 3.7% (median 2.7%), this ranged from 2.4% (median 2%) in region 1, to 4.6% (median 3.6%) in region 3. The overall mean rate of purchases into the herds was 18.6, (median 13.85), and this varied from 15.9 (median 13.1) (region 4) to 21.4 (median 16.6) (region 1) (Table 1). There were no clear geographic patterns observed between regions and between herds.

### Seroprevalence data

For BHV-1, the herd seroprevalence was 90% overall (Table 2, and this ranged from 76% in region 3 to 100% in Region 7. The mean within herd seroprevalence for BHV-1was 39.8%, and ranged from 11.1% in the Region 2 up to 45.5% in Region 7. Overall, 18% of herds were vaccinating for IBR, and this ranged from 11.5% in the Republic of Ireland to 45.5% in Northern Ireland. Results from the multivariate regression model indicated a positive association between BHV-1 seroprevalence and the herd prevalence of Neosporosis, the disclosure of BVDV positive animals within the BVD eradication scheme, herd size, and mortality rate (all age groups) (Table 3). There was a negative association between BHV-1 seroprevalence and calf mortality up to 30 days of age as well as with overall herd mortality.

**Table 2:** Herd sero prevalence and the percentage of herds vaccinating for BHV-1, the mean and median number of cows tested per herd, and the mean and median within herd BHV-1 sero prevalence

Region	% BHV-1 vaccination	BHV 1 Herd sero prevalence	BHV-1Within Herd Prevalence	
			Mean	Median
1	0	90	49.2	54.8
2	11.1	83	18.6	10.6
3	12.5	94	48.2	46.3
4	11.8	76	30.6	26.9
5	8.8	94	44.1	48.2
6	25.0	96	36.1	33.1
7	45.5	100	52.3	44.5
Overall	18.0	90	39.79	38.1

**Table 3:** A linear regression model of BHV-1sero prevalence with Neospora within herd prevalence, the disclosure of BVD virus positive animals in the herd, herd size, the number of animals dead per year, mortality rate, mortality rate up to 30 days as predictors which fit

	Coef.	Std. Err.	t	P > t	95% Conf. interval	
Neospora within herd sero prevalence	0.852	0.369	2.11	0.022	0.123	1.562
Disclosure of BVD virus positive animals in herd	11.466	5.249	2.18	0.031	1.083	21.851
Herd Size	0.139	0.049	2.84	0.05	0.042	0.236
Overall mortality	−2.416	0.977	−2.47	0.015	−4.350	−0.483
Mortality rate	3.716	1.325	2.80	0.006	1.095	6.337
Mortality rate up to 30 days of age	−0.186	0.076	−2.45	0.015	−0.337	−0.036
Intercept	15.654	6.649	2.35	0.020	2.501	28.807

Number of observations =139

F (6,132) = 4.3

Prob >F = 0.0005

R-square = 0.1636

Adj R- squared = 0.1256

Root MSE = 27.85

For BVDV, the herd seroprevalence was 100% in all regions (Table 4). The mean within herd seroprevalence of BVDV was 77.7%, and ranged from 42.8% in Region 2 up to 88.3% in Region 7. Overall, 32.3% of herds were vaccinating for BVDV, and this ranged from 23.5% in regions 4 and 5 to 57.1% in Region 1. The linear regression model for BVDV seroprevalence, showed associations with the seroprevalence of Neosporosis, the overall number of cattle that died in the herd in 2014, and the calves produced per cow in 2014 (Table 5).

**Table 4:** The herd sero prevalence for BVDV, the mean and median within herd BVDV sero prevalence, percentage of herds vaccinating for BVD, and the percentage of herds where a BVDV animal was disclosed

Region	BVDV Herd Sero prevalence	BVDV Within Herd Sero prevalence		% BVD vaccination	% herds with BVD virus positive calves
		Mean	Median		
1	100	71.7	68	57.1	50
2	100	42.8	37.7	38.9	28
3	100	87.8	93.7	25.0	50
4	100	81	98.1	23.5	18
5	100	69.3	72.9	23.5	23
6	100	80.5	88.4	32.5	35
7	100	88.3	96	36.4	NA
Overall	100	77.7	85.2	32.3	34

**Table 5:** A linear regression model of BVDV sero prevalence with Neospora canium within herd prevalence, the number of animals dead per year, and calves per cow per year as predictors which fit

	Coef.	Std. Err.	t	P > t	95% Conf. interval	
Neospora within herd sero prevalence	0.807	0.402	2.01	0.048	0.008	1.606
Overall mortality	1.550	0.561	2.76	0.007	0.435	2.665
Calves per cow per year	−30.431	13.401	−2.27	0.026	−57.059	−3.804
Intercept	93.377	12.341	7.57	0.000	68.856	117.898

Number of observations =93

F (3,89) = 4.50

Prob >F = 0.0055

R-square = 0.1317

Adj R- squared = 0.1025

Root MSE = 23.47

One or more BVDV virus positive animals were found in 46 of the 139 (34%) Republic of Ireland herds since the inception of the compulsory BVD programme in the Republic of Ireland (Table 4). The annual incidence of herds where BVDV positive animals were founded for 2013, 2014 and 2015 were 12.2%, 7.9% and 9.3%. The disclosure of BVD virus positive animals was associated with increasing herd size and increasing BHV-1seroprevalence (Table 6).

**Table 6:** The presence of BVD virus positive animals in the herd logistic regression model

	Odds ratio	Std. Err.	z	P > z	95% Conf. interval	
No cows in herd	1.025	0.010	2.62	0.009	1.006	1.045
BHV-1herd sero prevalence	1.013	0.006	2.02	0.044	1.000	1.026
Intercept	0.118	0.056	−4.51	0.000	0.046	0.298

No of observations =139

LR chi 2 (2) = 12.98

Prob > chi 2 = 0.0015

Pseudo R 2 = 0.0735

Log likelihood = −81.755

For Leptospirosis, the overall herd seroprevalence was 91%, and this ranged from 80% in Region 1 to 100% in Region 7 (Table 7). The mean within herd seroprevalence of leptospirosis was 65.7%, and ranged from 43.4% in Region 4 up to 73.3% in Region 6. Overall, 47.2% of herds were vaccinating for Leptospirosis, and this ranged from 23.5% in Regions 4 to 78.6% in Region 1. There was an association between the use of BVDV vaccine in the herd, the herd size and the purchasing patterns, particularly in 2014 and the Leptospirosis seroprevalence in the Leptospirosis linear regression model (Table 8).

**Table 7:** Herd seroprevalence and within herd sero prevalence and percentage of herds vaccinating for Leptospirosis

Region	Herd Sero Prevalence	Within Herd Sero Prevalence		% Leptosporisis vaccination
		Mean	Median	
1	80	79.2	77.7	78.6
2	83	70.4	81.4	50.0
3	94	68.2	68.8	31.3
4	88	43.4	45.9	23.5
5	94	72.6	83.3	52.9
6	96	73.3	78.6	45.0
7	100	60.7	72.2	50.0
Overall	91	65.7	75.4	47.2

**Table 8:** A linear regression model of leptosporosis sero prevalence with number of cattle purchased in marts and herd size as predictors which fit

	Coef.	Std. Err.	t	P > t	95% Conf. interval	
Herd vaccinating BVD	27.979	12.373	2.26	0.027	3.293	52.664
No of mart purchases in 2014	0.247	0.081	3.03	0.003	0.084	0.041
Herd size	0.093	0.029	3.21	0.002	0.035	0.151
Intercept	44.929	5.013	8.96	0.000	34.928	54.93

Number of observations =73

F (3,69) = 9.92

Prob >F = 0.000

R-squared = 0.3013

Adj R- squared = 0.2709

Root MSE = 23.942

For Neosporosis, the herd prevalence was 67% overall, and this ranged from 44% in Region 2 to 85% in Region 6 (Table 9). The mean within herd seroprevalence of Neosporosis was 5.7%, and ranged from 2.2% in Region 2 up to 7.4% in Region 1. Linear regression revealed a negative association between Neosporosis and both (i) the disclosure of BVDV positive animals in the herd during the BVDV eradication scheme and (ii) herd BHV-1seroprevalence (Table 10).

**Table 9:** Herd and within herd seroprevalences for Neospora canium

Region	Neospora herd sero prevalence	Within Herd Prevalence	
		Mean	Median
1	60	7.4	6.6
2	44	2.2	0
3	75	4.5	2.05
4	76	6.2	3.3
5	71	6	3.9
6	85	5.9	5
7	55	6.9	2.45
Overall	67	5.7	3.7

**Table 10:** A linear regression model of Neospora caninum sero prevalence with BHV-1sero prevalence and the disclosure of BVD virus positive calves as predictors which fit

	Coef.	Std. Err.	t	P > t	95% Conf. interval	
BHV-1Herd sero prevalence	0.045	0.018	2.45	0.016	0.008	0.082
Disclosure of BVD virus positive animals in herd	−2.355	1.171	−2.01	0.046	−4.671	−0.040
Intercept	4.531	0.907	5.00	0.000	2.739	6.325

Number of observations =139

F (2,136) = 4.18

Prob >F = 0.0173

R-squared = 0.0579

Adj R- squared = 0.044

Root MSE = 6.357

## Discussion

This study describes the herd level characteristics of a large scale study of over 6000 beef cows from 161 herds and is the first study of its kind to be conducted across the island of Ireland. The serological sampling and analysis for the four pathogens of interest was carried out specifically for the purposes of the study. To the knowledge of the authors, no previous study has concurrently examined multiple pathogens in the same beef cows, which facilitates the study of potential associations amongst these pathogens on economically important outcomes at herd level. As part of our study we combined seroprevalence data, a targeted brief herd questionnaire, data on the BVDV status of each herd from the ICBF database, and animal identification and movement data (AIM data) for the first time within an Irish context. This is one of the first studies in Europe to integrate data from the national cattle identification and movement database with disease prevalence data, to identify risk factors associated with the occurrence of BHV-1, BVDV, Leptospirosis and Neosporosis.

Increasing herd size emerged as a factor associated with seroconversion to BHV-1, BVDV and Leptospirosis, and the disclosure of BVDV positive animals. Herd size has previously been documented as a significant risk factor associated with exposure to these pathogens in Ireland [5, 11, 14, 20]. Similar experiences have been documented internationally for several pathogens. Increasing herd size increases the probability of an individual animal becoming exposed to pathogens and becoming a carrier animal, and is a well recognised risk factor for the occurrence of disease. Larger herds are likely to have management practices which could predispose them to exposure to the pathogens. Additionally the purchase of animals into the herd is often required to achieve increased herd size but this too is an acknowledged risk factor associated with exposure to pathogens.

There were numerous associations identified between the pathogens under investigation. For example, the seroprevalence of BHV-1was associated with the seroprevalence of Neosporosis, and the disclosure of BVDV positive calves in the herd. Indeed, the role of co-infection has been previously described by Van Leeuwen et al. [15], where cows seropositive for Bovine Leukaemia Virus (BLV) were 1.5 times more likely to be seropositive for Neosporosis than BLV seronegative cows. Another possible explanation is that infection with one of the pathogens could facilitate infection with another. The role of BVDV, in particular, as an immunosuppressive agent is well documented [4].

### BHV-1

In addition to herd size and associations with other pathogens increased BHV-1 seroprevalence was associated with increased overall herd mortality rates. Previous studies have reported herd level seroprevalences of 77% and 74% in Northern Ireland and the Republic of Ireland, respectively [5, 20]. The 90% BHV-1 herd prevalence reported in the present study would indicate that BHV-1 is endemic in the Irish cattle population. The level of herd prevalence described here is probably related to the relatively large size of the participating herds. An earlier study also found a significant association between BHV-1 seroprevalence and herd size in Ireland [11]. Previous studies have reported IBR vaccine usage of 1.8% and 11% in the Republic of Ireland and Northern Ireland respectively [11, 20]. The comparable levels of IBR vaccination in the present study, were 11.5% and 45.5% respectively, which is a marked increase on the previous studies by Cowley et al., [11, 20]. This may reflect a greater awareness of animal health issues among the owners of our study herds employed here, which included research herds and herds participating in Knowledge Transfer demonstration programmes.

### BVDV seroprevalence

Previous studies have described herd level seroconversion to BVDV in excess of 98% in both the Republic of Ireland and Northern Ireland [5, 20]. Similar levels of herd seroconversion to BVDV have been reported in England and Wales [21]. In our study 32% of herds were vaccinated for BVD, which is substantially greater than the rates of 2.2% in the Republic of Ireland and 16% in Northern Ireland reported in studies of beef herds [5, 20]. We found evidence of an association between seroprevalence of both BVDV and Neosporosis. BVDV seroprevalence was also positively associated with the total herd mortality (2014) in the herd. There was a negative association between the calves produced per cow per year (2014) and increased BVDV seroprevalence in the herd. Both of these findings are not unexpected as BVDV infection is associated with increased calf morbidity, increased mortality as well as early embryonic death [22], which would decrease the number of calves per cow per year.

### Disclosure of BVD virus positive calves

The disclosure of BVDVpositive calves was related to the seroprevalence of BHV-1and herd size. One third of the Republic of Ireland study herds participating in the national BVD eradication scheme reported one or more BVD virus positive calves in the initial 3 years of the scheme [23]. The disclosure of BVDV positive calves in many of the herds in this study could account for the high levels of herd and within herd seroconversion observed. Herd size was a significant risk factor in this study which is consistent with previous Irish reports [24]. It is of note that the disclosure of BVDV positive calves in the herds in this study was consistently higher than herds in the general population as recorded as part of the Irish national BVD eradication scheme [23].

### Leptospirosis seroprevalence

In addition to herd size, the seroprevalence of Leptospirosis was associated with the level of livestock mart purchases in 2014. It is difficult to determine the significance of this, as it was only found to reach statistical significance in 2014. This association is likely to be biosecurity related, and was associated with movement of animals between holdings and spread within holdings.

In this study there was evidence of seroconversion among 90% of non-vaccinating herds, which was greater than the 82% herd level seroprevalence described in a previous Irish study of beef cow herds [14] (Table 7). The mean within herd seroprevalence was 75%, which was almost twice the rate of the 40% seroprevalence described by Ryan et al., [14]. That study concluded it was likely that there were carrier animals actively transmitting leptospires at the seroprevalences described in their study. They also described a regional effect in relation to Leptospirosis seroprevalence, although no such pattern was observed in our study. They also reported a positive association between herd seroprevalence and herd size. Previously, Leptospirosis was described as independent of region and rainfall [25]. A previous UK study has described a 24.2% seroprevalence in beef herds [26]. The high within herd seroprevalence in our study is probably related to the fact that beef calves spend relatively more time in contact with adult cows than do dairy calves and there is consequently a greater risk of exposure to carrier animals in the herd. In addition, this contact is greater during winter housing, where calves tend to be kept on creep areas and are able to enter a pen of calved cows, some of which may be carriers. In this study, almost half of herds (47%) were vaccinating for Leptospirosis, which is substantially greater than the 3% described by Ryan et al., [14].

### Neospora seroprevalence

The herd seroprevalence for Neosporosis of 67% reported here is almost identical to that of a similar study in Australian beef cow herds [19], which also reported a within herd seroprevalence similar to our findings. The within-herd prevalence of Neosporosis was relatively low compared to the other three pathogens studied, which probably reflects the fact that transmission of Neosporosis, is, for the most part vertical [27]. A previous US study found prevalence was lower among beef cows grazed on pasture as opposed to housed cows [28]. This might suggest that horizontal transmission, or exposure from a point source, could account for herds with a higher within herd seroprevalence. Exposure to *Neospora caninum* has previously been associated with the presence of dogs and wild canine species such as foxes on the farm [15, 16]. In our study, participants were not asked about the presence of dogs on the farm, but it is worthy of further consideration. The seroprevalence of Neosporosis was associated with the seroprevalence of BHV-1and the disclosure of BVDV positive animals in the herd. Seroconversion to Neosporosis has also previously been linked to the presence of BVDV exposure in herds [15].

The farmers that participated in our study volunteered to do so and were not evenly distributed across the island of Ireland. Region did not emerge as a significant variable in any of the multivariate analyses and the disease prevalences in the various regions were broadly similar. While this study may not be representative of beef cow herds overall, participating herds came from a wide range of sizes, which enabled us to adequately investigate the effect of herd size.

While there are few perfect serological tests available, the tests that were used in this study were of a high sensitivity and specificity. We are confident that the tests BHV-1, BVDV and Letporosis provided areflection of the sero status of the animals tested. However, given the dynamics of the host parasite interaction and the characteristics of neospora serological tests, it is probable that the Neospora ELISA underestimated the true prevalence of Neosporosis in the cows tested [29].

The level of seroconversion to BHV-1in Northern Ireland may have been over estimated, as conventional non marker BHV-1vaccines are still available on the market there. For this reason, data from herds located in Northern Ireland were not included in the linear model, and the BHV 1seroprevelance in the various regions was calculated individually as we avoided using a cumulative figure so as to prevent any one region biasing the overall results.

Seroconversion is a reflection of historic exposure to a particular pathogen, and is not necessarily an accurate reflective of infection status of an individual animal. However, a high herd seroprevalence, especially among younger animals in a herd, may be suggestive of active infection in that herd. This study was herd based and could not take account of the age of the animals within those herds. The issue of individual animal factors will be addressed in a parallel piece of research (Parr et al., unpublished). Only seropositive animals were included in calculating the herd seroprevalence, and animals with an inconclusive test result were treated as seronegative as this was a herd level based study.

Vaccine usage was much higher among herds in this study than in previous studies. This may be a reflection of the targeted nature of participant selection, whereas previous studies selected herds randomly. The farmers in our study volunteered for inclusion and were therefore likely to be more motivated than the general population.

## Conclusions

Our findings support those of several previous studies that as herds increase in size there is increased exposure to pathogens. Consequently farmers with larger herds need to be aware that they must increase their efforts to avoid the introduction and spread of pathogens to their herds, to establish the health status of their herds and determine what mitigation measures are required to address those diseases.

This study has also shown that exposure to several pathogens is associated with exposure to other pathogens. This suggests that there is a common factor common to the exposure among these herds, which is likely to be related to biosecurity. This also indicates that biosecurity needs greater attention in larger herds.

## Abbreviations

AIM: Animal Identification and Movement database
BHV-1: Bovine Herpes Virus - 1
BLV: Bovine Leukaemia Virus
BVD(V): Bovine Viral Diarrhoea (Virus)**,**
HPRA: Health Products Regulatory Authority
IBR: Infectious Bovine Rhinotracheitis
ICBF: Irish Cattle Breeding Federation
